# Effect of static seeding methods on the distribution of fibroblasts within human acellular dermis

**DOI:** 10.1186/1475-925X-12-55

**Published:** 2013-06-24

**Authors:** Mario Vitacolonna, Djeda Belharazem, Peter Hohenberger, Eric D Roessner

**Affiliations:** 1 Division of Surgical Oncology and Thoracic Surgery, Department of Surgery, University Medical Centre Mannheim, Mannheim, Germany; 2 Institute of Pathology, University Medical Centre Mannheim, Mannheim, Germany

## Abstract

**Introduction:**

When developing tissue engineered solutions for existing clinical problems, cell seeding strategies should be optimized for desired cell distribution within matrices. The purpose of this investigation was to compare the effects of different static cell seeding methods and subsequent static cell culture for up to 12 days with regard to seeding efficiency and resulting cellular distribution in acellular dermis.

**Materials and methods:**

The seeding methods tested were surface seeding of both unmodified and mechanically incised dermis, syringe injection of cell suspension, application of low-pressure and use of an ultrasonic bath to remove trapped air. The effect of “platelet derived growth factor” (PDGF) on surface seeding and low pressure seeding was also investigated. Scaffolds were incubated for up to 12 days and were histologically examined at days 0, 4, 8 and 12 for cell distribution and infiltration depth. The metabolic activity of the cells was quantified with the MTT assay at the same time points.

**Results:**

The 50 ml syringe degassing procedure produced the best results in terms of seeding efficiency, cell distribution, penetration depth and metabolic activity within the measured time frame. The injection and ultrasonic bath methods produced the lowest seeding efficiency. The incision method and the 20 ml syringe degassing procedure produced results that were not significantly different to those obtained with a standard static seeding method.

**Conclusion:**

We postulate that air in the pores of the human acellular dermis (hAD) hinders cell seeding and subsequent infiltration. We achieved the highest seeding efficiency, homogeneity, infiltration depth and cell growth within the 12 day static culturing period by degassing the dermis using low- pressure created by a 50 ml syringe. We conclude that this method to eliminate trapped air provides the most effective method to seed cells and to allow cell proliferation in a natural scaffold.

## Introduction

There is significant interest in engineered soft tissue implants that could extend the range of defect filling options available to surgeons. The majority of these procedures use autologous donor sites as a source of soft tissue, whereas the donor site creates a further wound that increases recovery time, morbidity, loss of function and the risk of infection [1,2]. The reconstruction of tissue with tissue engineered constructs based on a suitable scaffold seeded with autologous cells could partially mitigate this problem [3,4]. Such a scaffold should ideally mimic the extracellular matrix (ECM) of native tissue to facilitate optimal growth and differentiation of cells, be stable, easy to use, biodegradable and biocompatible. Acellular dermis transplants fulfill most of the prerequisites of a natural, stable scaffold [5-7]. Such biological scaffolds from decellularized tissues have been successfully applied in animal and clinical studies [7-13]. A critical step is the seeding of cells into the scaffolds. Effective cell seeding can be critical for the successful clinical application of tissue-engineered products [14]. It is therefore important to obtain an optimal distribution of the cells within the matrix. Over the last few years numerous papers on this topic have been published. However, most of these dealt with synthetic or biological scaffolds based on reconstituted collagen [15-18]. The porosity of such matrices can be controlled during manufacturing, whereas transplants derived from native tissues have a predetermined matrix structure that may be more difficult to seed appropriately. Cells can be more easily dispersed in open network structures than in the more dense protein networks generally found in natural tissues [5,6]. Nevertheless, natural matrices can have potential advantages over synthetic matrices since additional important extracellular matrix components may be present. The presence of growth factors may, for example, accelerate revascularization [19-21].

The purpose of this investigation was to compare different static cell seeding methods for their seeding efficiency, homogeneity and infiltration depth using an human acellular dermis (hAD, Epiflex®, DIZG, Germany) [12].

The extent to which cell seeding processes can be sustained in vitro depends on the cell type, the nature of the scaffold and associated transport conditions. We investigated our seeding system in this respect by examining the distribution and metabolic activity of the cells over a 12 day culture period.

We used fibroblasts derived from subcutaneous fat in our study, since a cell-seeded dermis might serve as a graft for soft tissue defects where such cells are important. It is known that these cells secrete cytokines during the wound healing process [22].

## Materials and methods

### Cell isolation and culture conditions

Fibroblasts were obtained from the subcutaneous fat of male Fischer-344 rats weighting 250–300 g (Charles River), as previously described [23]. The adipose tissue was digested using 2 mg/ml collagenase type 2 (PAA, Germany) at 37°C for 2 h with vigorous shaking. The suspension was washed twice with Dulbecco’s modified Eagle medium (DMEM) with 4,5 g/L glucose (PAA, Germany) containing 10% (v/v) fetal bovine serum (FBS) (PAA, Germany) and the suspension was centrifuged at 400 g for 5 min. The resultant cell pellet was plated on 100 mm^2^ tissue culture plates (Greiner Bio One, Germany) with DMEM (supplemented with 10% FBS and 1% penicillin/strepavidin solution (PAA, Germany)) and maintained at 37°C in an incubator with 5% CO_2_. The medium was changed every 3 days and passages were carried out at 70% to 80% confluence. Only cells from passage 7 were used for the subsequent seeding procedures.

### Standard seeding and cell culture

Human acellular dermis with a thickness of <0.8 mm were cut in 10×10 mm pieces and rehydrated in DMEM for 2 h at 37°C in 48 well plates (Greiner, Germany). Fibroblasts were seeded onto the hAD pieces (n = 10/method) with the appropriate method at a concentration of 4×10^5^ cells/hAD and a volume of 300 μl. Culture plates were incubated after the corresponding seeding procedure at 37°C with 5% CO_2_ for 2 h to enable cell attachment. The seeded scaffolds were then transferred to new 48 well plates, and 300 μl of DMEM was added to each well. Plates were incubated at 37°C and medium was changed daily. After seeding, hADs were incubated for up to 12 days and analyzed at day 0, 4, 8 and 12 to determine the effects of the seeding methods. At each time point, 4 seeded scaffolds were fixed, embedded vertically in paraffin, histologically sectioned and stained with propidium-iodide (PI) (Molecular Probes, USA) to visualize the cell distribution and penetration depth. Furthermore the metabolic activity of 6 hADs was evaluated by MTT assay as an index of proliferation and seeding efficiency as described below.

### Scaffold seeding methods

Static surface seeding was performed as described above. Cell suspension injection was performed with a modification of a previously described method [24]. A cell solution with 4×10^5^ cells/hAD was injected into 9 points uniformly distributed over the scaffolds using a 25-gauge needle. Subsequently, the scaffolds were transferred into new wells and 300 μl fresh medium was added.

The incision method was performed by making a series of 9 small full thickness cuts of approximately 2 mm length uniformly distributed over the scaffold surface using a scalpel. Subsequently, the matrices were seeded as in the static seeding method. After the 2 h incubation at 37°C, the scaffolds were transferred into new wells and 300 μl fresh medium was added.

The low-pressure seeding environment was established with a 20 ml or 50 ml syringe (Becton Dickinson, USA) and a stopcock as described elsewhere [25]. Briefly, the scaffolds and 5 ml of DMEM were aseptically transferred to the syringe, so that the matrices were immersed in the liquid, closed with the plunger and an additional 5 ml of air was drawn into the syringe. The syringe was then closed with a stopcock. To create a vacuum, the plunger was pulled back to the end of the syringe, and maintained and shaken in this position for 60 s to remove the air bubbles from the matrices. After 60 s the stopcock was opened abruptly to burst the air bubbles. Manometric measurements with a PCE-917 pressure gauge (PCE- Inst., Germany) using this procedure demonstrated, that the maximum negative pressure that could be generated with a 20 ml syringe, was about 62.5 kPa and with a 50 ml syringe about 75.5 kPa. This procedure was repeated 5 times. Then, the scaffolds were transferred into a 48 well plate and subsequently seeded with the static method. After the appropriate method and 2 h attachment at 37°C, the scaffolds were transferred into new wells.

For the method using the ultrasonic bath, the matrices were immersed in a 50 ml tube that was filled with 20 ml medium. The tube was placed in an ultrasonic bath for 30 minutes. After this procedure, the matrices were seeded and treated according to the static seeding method.

Two subgroups (static seeding and 50 ml syringe degassing) were additionally treated with PDGF (Upstate Biotechnology, USA). The mitogen was added daily at a concentration of 50 ng/ml.

### MTT assay and calculation of seeding efficiency

To determine the efficiency of the cell-seeding methods, metabolic activity was evaluated with the MTT assay [26]. MTT (Sigma Aldrich) was dissolved in PBS at a concentration of 5 mg/ml. Culture media was replaced with 270μl DMEM and 30 μl of MTT solution (1:10 dilution) was added to each well. After 4 hours incubation at 37°C, scaffolds were placed into new wells and cells were lysed with 500 μl lysis buffer (1g SDS; 60 μl acetic acid; 9,94 ml DMSO) overnight at room temperature on a plate shaker. Extraneous fluid was then carefully expressed from the matrices and the supernatant of each well was transferred into a new well. Optical density (OD) was measured in a microplate reader (Biochrom Anthos, Biochrom, Germany) at 570 nm with a 630 nm reference filter. 6 hAD were evaluated for each of the seeding methods.

Relative seeding efficiency was deemed to be represented by the fraction:

$$\mathrm{OD}\;\left(\mathrm{MTT}\right)\;\mathrm{seeding}\;\mathrm{method}/\mathrm{OD}\;\left(\mathrm{MTT}\right)\;\mathrm{reference}\;\mathrm{method}\;\left(\mathrm{static}\right).$$

The absolute seeding efficiency of the static method was evaluated according to a modification of a previously published method [24]: Six wells containing 4×10^5^cells/well and a test matrix were incubated at 37°C for 2 h. Thereafter the matrix was turned over in the well and the previous incubation step was repeated. These wells were then subjected to the MTT assay without transferring the matrix to a new well. Thus both the cells adhering to the matrix and those adhering to the well itself were included in this analysis. The absolute seeding efficiency (%) of the static method was then deemed to be represented by the fraction:

$$\mathrm{OD}\;\left(\mathrm{MTT}\right)\;\mathrm{reference}\;\mathrm{method}\;\left(\mathrm{static}\right)/\mathrm{OD}\;\left(\mathrm{MTT}\right)\;4\times 10^{5}\mathrm{cells}.$$

### Cell distribution and penetration depth

To analyze the cellular distribution histologically, hADs were fixed in 10% formalin for 24 hours and sliced into 3 equal parts. Each of them were then embedded vertically in paraffin and histologically sectioned (7 μm) with a microtome (Microm, Germany) for a complete edge-to-edge cross-sectional view. Samples were mounted onto glass slides, dried overnight at 37°C, deparaffinized by submerging them 3 times for 5 min in a xylene bath and subsequently 3 times for 2 min in each of a series of dilutions of ethanol (100%, 96% and 80%). Thereafter, the sections were incubated with Fluoroshield® containing PI (Sigma Aldrich, USA) to stain the cell nuclei. Digital images of the whole edge-to-edge cross-section were obtained using an ×10 objective and a fluorescence microscope (Zeiss, Germany) fitted with an appropriate filter. Images were stitched with ICE (Image Composite Editor, V1.4.4, Microsoft, USA). To quantify the extent of infiltration, the cross sections were considered to consist of 3 layers (Zone 1 (10-150 μm from the upper surface), Zone 2 (151-300 μm from the upper surface) and Zone 3 (>300 μm from the upper surface)) and the number of cells found in each zone was recorded as a percentage of the total number of cells observed in each section.

**Figure 1:**
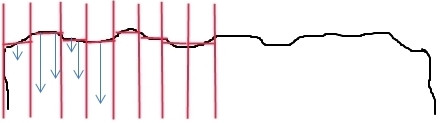
**Scheme of the surface idealization of the hAD.** The red lines indicate the subdivision of the area and the idealized surface; the blue arrows indicated measured lengths.

Since the hAD surface showed strong undulations, the extent of cellular infiltration was considered in each of a series of idealized compartments as depicted in Figure 1. For each of these compartments a normalized surface was considered and the extent of infiltration was considered to be the average perpendicular distance of cells from this normalized surface. This distance was measured with AxioVision Software (Zeiss, V4.8, Germany). Only those cells at least 10 μm from this idealized surface were included in the analysis. Compartments at the edges of the sections were not included in the analysis. 4 hAD per seeding method were sectioned and 5 sections from each hAD were evaluated.

### Statistics

The effect of each treatment was statistically analyzed by pairwise comparisons of the treatments by means of non-paired t-tests with significance set at the 95% confidence level. All values are expressed as means ± standard deviation of the mean (SD). All statistical analyses were performed by a qualified statistician from the University Hospital Mannheim using SAS (Version 9.2, SAS Institute).

## Results

### Seeding efficiency

**Figure 2:**
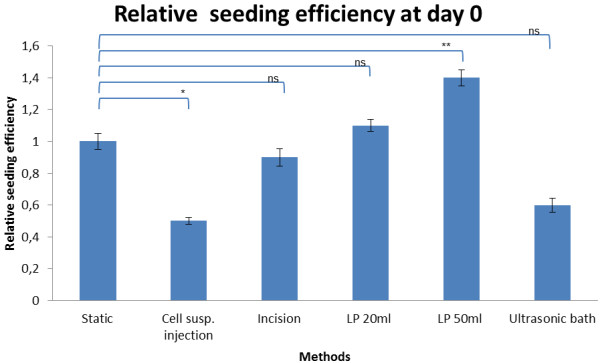
Relative seeding efficiency 6 h (2 h of incubation to allow cells to adhere and 4 h incubation to allow reaction with MTT) after the appropriate seeding procedure (normalized raw data from MTT Assay (OD Method / OD Static Method).

A summary of the results is provided in Table 1 and Figure 2. In each analysis, one seeding method is compared with the static method. The OD of wells in which all cells (both matrix-associated and those attached to the culture surface of the plates) was .14 ± 0.05. The OD in wells with matrices seeded with the standard method was 0.47 ± 0.05. The absolute seeding efficiency of the static method was therefore 42%.

**Table 1:** The relative seeding efficiencies after 6 hours of incubation expressed as a factor of that of the static method

**Methods**	**Seeding efficiency /%**	**Factor**	**OD**	***P *value**
Static	**42**	**1**	**0.47 ± 0.05**	
Cell suspension injection	20	0.5	0.23 ± 0.02	p = 0.0001
Incision	41	1	0.47 ± 0.05	p = 6
Low-pressure 20ml syringe	45	1.1	0.52 ± 0.04	p = 0.2196
Low-pressure 50ml syringe	57	1.4	0.64 ± 0.05	p = 0.0033
Ultrasonic bath	24	0.6	0.27 ± 0.04	p = 0.6505

The values are derived from the raw data from the MTT Assay (OD). The p-values are those derived from pairwise comparisons with the static method (T-test).

As can be seen in Table 1, a significant increase in seeding efficiency was observed when the degassing step using a 50 ml syringe (1.4×; p = 0.0033) preceded static seeding. Furthermore, when the scaffolds were degassed with the 50 ml syringe method, the number of cells retained in the matrix was 0.3x significantly higher than that recorded with the 20 ml syringe method (p = 0.0071). Injection of the cell suspension and use of the ultrasonic bath for degassing, resulted in seeding efficiencies that were respectively 0.5 and 0.6 those of the results with the static method. The incision method and degassing with a 20 ml syringe produced results that were not significantly different than those from the standard method.

### Cell distribution and penetration depth after seeding

**Figure 3:**
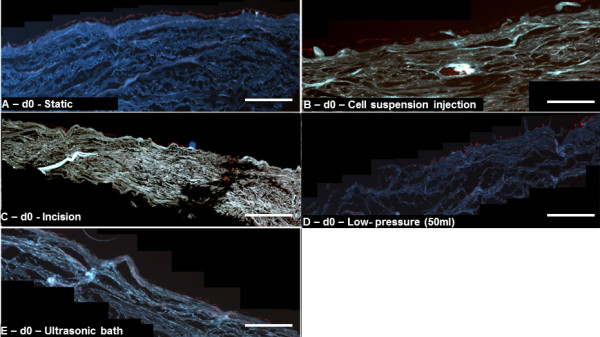
**Representative immunofluorescence cross section images of seeded matrices (blue) after seeding.** Fibroblast DNA was stained with PI (red) to visualize the cellular distribution. (**A**) Static seeding, (**E**),ultrasonic bath (**D**) and low pressure (50 ml syringe) resulted in a monolayer of cells on the surface of the scaffold, while (**B**) cell suspension injection and incision (**C**) resulted in localized cellular aggregation. Scale bars are equal to 1 mm.

Table 2 and Figure 3 show a summary of the results. Static seeding resulted in a thin cell monolayer on the surface of the hAD with no penetration into the scaffold (Figure 3A). When using the injection seeding method, only dense clusters of cells were found in the vicinity of the original injection sites (Figure 3B).

**Table 2:** Cellular penetration and distribution in the matrices

**Methods**	**Penetration depth/ μm**	**Total number of penetrated cells per idealized compartement**	**% cells**	**% cells**	**% cells**
			**10-150 μm**	**151-300 μm**	**>300 μm**
Static seeding	0	0	0	0	0
Incision	148.1 ± 85.1	27 ± 14	50	46	4
Low-pressure (LP) 20 ml	82.3 ± 45.9	16 ± 8	97	3	0
Low-pressure (LP) 50 ml	118.3 ± 42.6	33 ± 12	76	24	0
Ultrasonic bath	0	0	0	0	0

Penetration depth (μm) is defined as the mean distance of a cell from the idealized surface. Penetration information is provided as the number of cells per idealized compartment in each of three depth zones (10–150 μm, 151-300 μm and >300 μm from the idealized surface).

The results from the incision method were similar to those obtained with the injection method. Here, a cell monolayer was found on the surface and clusters of cells with an average penetration depth of 148.1 μm ± 85.1 μm were observed. The distribution of these cells was 50% in Zone 1, 46% in Zone 2 and 4% in Zone 3. The average number of cells per idealized compartment with the incision method was 27 ± 14 (Figure 3C).

When using the 20 ml syringe we counted 16 ± 8 cells per idealized compartment. There were significantly fewer cells inside the matrix (p = 0.0011) and a reduced penetration depth (82.3 μm ± 45.9 μm) (p = 0.0314) compared to that observed with 50 ml syringe degassing (33 ± 12 cells/idealized compartment and 118.3 μm ± 42.6 μm) was recorded (Figure 3D).

Using the ultrasonic bath, no cells were detectable within the matrix and only a thin cell layer was present on the surface (Figure 3E).

### Cellular viability during cultivation

**Figure 4:**
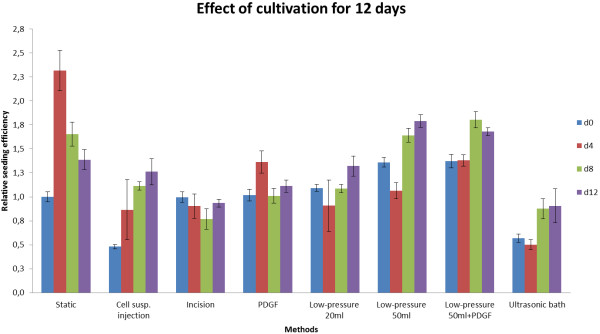
Effect of cultivation for 12 days. (normalized raw data from MTT Assay (OD method/OD static method).

The data are provided in Figure 4.

There appears to be an initial proliferative phase. This phase extends for all methods until at least day 4. For a subset of methods the proliferative phase seems to be longer. We therefore decided to conduct a statistical comparison of OD values (as an index of proliferation/survival) at day 8.

The ODs resulting from the 50 ml syringe degassing method were significantly higher than those from the standard method at day 8. This was the case for both PGDF-treated (p = 0.0004) and PGDF-free (p = 0.0009) cultures.

In general we observed the trend, that OD values of each group had a maximum proliferation at day 4 and thereafter a monotonic decrease until day 12 (Figure 4).

**Figure 5:**
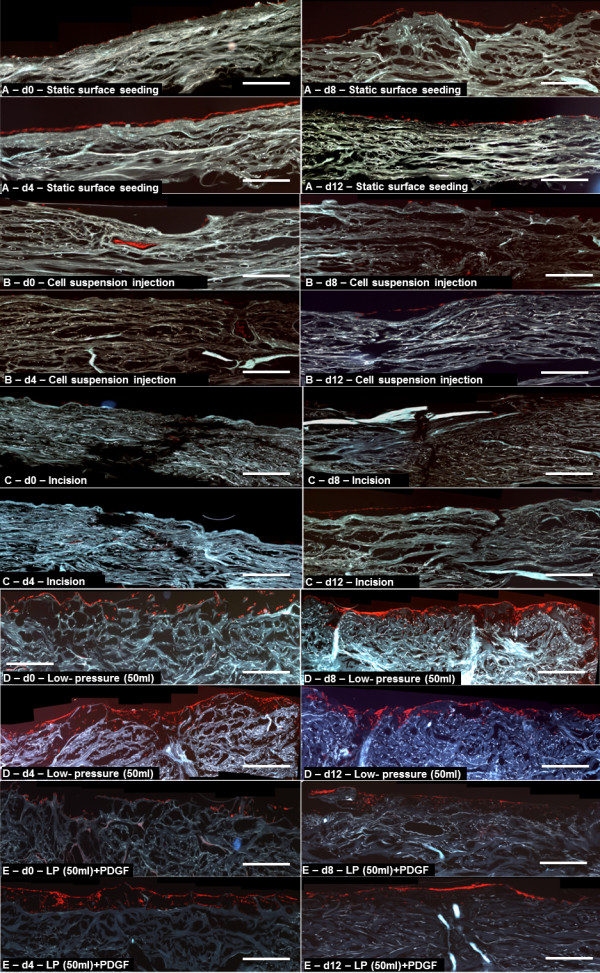
**Representative immunfluorescence cross section images of seeded matrices (blue) at day 0–12.** Fibroblasts were stained with PI (red) to visualize the cellular distribution. (**A**) Static seeding showed a thin cell layer on the surface with a maximum at day 4 and thereafter a continuous decrease until day 12. (**B**) Local cell clusters were seen after the injection of the cell suspension, but these were lost by day 8. (**C**) Cell aggregates could be observed at the incision points but these were gradually lost over the culture period (**D**) Degassing with the 50 ml syringe method led to an increased proliferation of multiple layers near the surface of the dermis. (**E**) Degassing with the 50 ml syringe method combined with PDGF supplementation led to an initial increase in proliferation, but also to an earlier onset of cell death. Scale bars are equal to 1 mm.

The static seeding method showed no infiltration, only a cellular monolayer on the surface that reached a maximum at day 4 and decreased significantly until day 12 (Figure 5A).

The cell clusters created by the local injection of cell suspension were barely detectable after 8 days. From this point only a sparse surface layer, probably propagated by a few cells that adhered to the surface during the incubation was visible (Figure 5B). The OD values were not significant higher than the values from the static method.

Similarly, cells seeded with the incision method could be observed only as local clusters inside the hAD. These displayed proliferation until day 12 (Figure 5C). At no time point was the proliferation greater than that of cells seeded with the static method.

Supplementation with PDGF resulted in a significantly increased proliferation at day 4 (p = 0.0159), but at day 8 and day 12 the cell numbers were significantly reduced compared to day 4. PDGF had no effect on penetration.

Degassing the hAD with the 20 ml syringe low pressure method, resulted in only a small number of penetrating cells and the OD values from day 4, 8 and 12 were not significantly higher than those of the static seeding method. Significantly better results were obtained using a 50 ml syringe to generate a low-pressure environment for degassing. As Figure 5D shows, starting from day 4 a dense infiltration and growth into the matrix was detectable and the cells grew until day 12 can be seen in Figure 4. Compared to all other methods, a significant higher OD could be reached at day 8 and 12 (p = 0.0009 and p = 0.0004).

Combining low pressure (with a 50 ml syringe) with PDGF resulted in a significant increase of proliferation compared with day 4 to 12 of the static method. However, no effect was observed on cell penetration. Cell numbers steadily declined after day 4 as can be seen in Figure 5E.

Using an ultrasonic bath resulted in reduced proliferation throughout the cultivation period when compared to the static method.

## Discussion

### Seeding efficiency and infiltration depth

The most commonly used method to seed a scaffold is static seeding. A cell suspension is deposited on the scaffold surface and the cells are allowed to infiltrate the scaffold [27]. This technique is simple, fast, with minimal technical requirements, feasible in almost any laboratory, and hydrodynamic damage to the cells can be expected to be minimal [28]. Nevertheless, this method seems to have potential disadvantages that may result in limited penetration, inhomogeneous distribution and inadequate seeding efficiency [29,30]. These limitations are a consequence of reliance upon diffusion and gravity for seeding.

In addition to the intrinsic problem of seeding matrices with cells, the requirement to quantify the distribution of cells throughout the matrix presents additional difficulties. We reviewed available techniques and decided to use the MTT assay to provide an index of metabolic activity and hence viable cell number. We evaluated proliferation over a 12 day incubation period and the extent to which cells were retained by matrices according to the individual seeding methods. We also measured the penetration depth and quantified the number of cells within defined zones via histology.

The pure static seeding method of deposition of the cell suspension on the surface of the dermis served as the reference method. With this method there was effectively no cellular infiltration. A thin layer of viable cells was observed on the surface of the hAD.

Injection of the cell suspension resulted in clusters of cells in the vicinity of the injection point. A uniform distribution of cells in the matrix is likely to be advantageous, since cell-cell signaling is necessary to generate functional tissue. An additional disadvantage of the injection method is the lack of a surface layer. Such a layer can be an important interface between the transplant and the wound bed.

The incision method also had disadvantages. Although a surface layer was formed, cellular infiltration was localized at the incision points. This method was devised as a means of increasing the surface area for cellular attachment. Published studies have demonstrated an increased in-vivo endothelialization with laser-perforated decellularized matrices [31,32]. However, we were not able to increase the efficiency of seeding with this method.

Low-pressure degassing alone produced a significant increase in efficiency in comparison to static seeding. The removal of trapped air from the dermis seems to enable the cells to penetrate deeper inside the scaffold during the 2 hour adhesion phase than was possible using the static seeding method.

This effect could not be achieved with the use of an ultrasonic bath for degassing the hAD. Use of an ultrasonic bath for degassing might be more practical than the syringe method. Therefore, we were interested in whether an ultrasonic bath could be used to degas the dermis. However, this method produced the lowest seeding efficiency. Although the microscope images revealed no evidence of structural damage, we hypothesize that the ultrasonic waves may have damaged matrix components such as collagens, laminin, or fibronectin or cell binding sites [33]. This method seems therefore to be unsuitable.

### Static cultivation

We cultured the seeded matrices for 12 days to determine how the seeding methods influence cell proliferation and repopulation of the dermis.

For this purpose, cell proliferation was assessed with an MTT assay at 4 day intervals. To exclude effects of nutrient exhaustion, the medium was changed daily. As seen in Figure 4, the OD values for most methods showed a maximum on day 4 and then a steady decrease between days 4 and 8. This effect is likely to be a consequence of increasing cell numbers and therefore increasing nutrient consumption leading to diminishing nutrient availability. Cell aggregation and associated phenotypic changes can have negative effects on the proliferation of cells in matrices which may invoke apoptosis [34-36]. After 4 days immunofluorescence microscopy revealed a dense layer on the surface that was one to three cells thick. Such a layer will result in a large reduction in dissolved oxygen partial pressure in the culture medium deeper in the matrix.

With the exception of degassing with a 50 ml syringe, the examined methods showed a continuous growth of cells until confluence was reached at the surface on day 4 and thereafter a progressive loss of cells. The incision method whilst increasing the surface area did not lead to a significant increase in proliferation, but rather led to major loss of cells after the proliferative phase. This effect may be explained by more pronounced formation of cell aggregates and consequent loss of proliferative function and apoptosis. When injecting the cell suspension, the same problem was observed.

Since a published report suggested that PDGF can increase cell proliferation in such environments [37], we decide to investigate the mitogenic effect of PDGF in our test system. We recorded only minimal early influence of PDGF and we therefore consider its use in clinical application of seeded matrices to be questionable. From a regulatory standpoint avoiding use of such compounds is advisable.

It has been argued that after rehydration of natural matrices such as acellular dermis, a large volume of air is present in the pores of the matrix [38]. It seems to be particularly important for recellularization to remove the air pockets inside the matrices [39]. Trapped air may, in addition to blocking pores, also lead to cell membrane damage. The use of a 50 ml syringe in comparison to a 20 ml syringe increased the cell mass and penetration depth significantly. It is conceivable that through variation and optimization of the degassing procedure higher values could be achieved [39]. In addition, removal of air could also lead to an accelerated growth of blood vessels [40].

As we demonstrated with our study, using conventional static seeding methods, cells grow mostly as a superficial layer on the surface of the scaffold. During the 12 days in-vitro cultivation, we could not detect an extensive penetration of viable cells into the deeper layers of the dermis. To maximize diffuse transport, it seems to be important, to remove air bubbles which are remnants of the rehydration process within the matrix to maximize penetrable volume. Removing air bubbles by degassing with a 50 ml syringe, we were able to achieve the highest seeding efficiency as well as the highest and most consistent increase in cell mass within the 12-day cultivation period.

## Conclusions

The present study demonstrates that a pre-seeding, low pressure degassing step significantly increased the proliferation of dermal fibroblasts in an acellular dermis tissue transplant. Although cellular penetration was limited, we anticipate that post-transplantation revascularization could be supported and enhanced by the fibroblasts located on or near the surface of such a seeded transplant. This revascularization should in turn support deeper repopulation by fibroblasts and thus maybe a faster healing and integration after transplantation.

## Competing interests

The authors declare that they have no competing interests.

## Authors’ contributions

The study was conceived and designed by MV, ER and PH. The experiments were conducted and the results analyzed by MV and DB. The manuscript was written by MV, DB, ER. All authors read and approved the final manuscript.
